# Endoscopic ultrasound – fine needle aspiration of 2-deoxy-2-[^18^F] fluoro-D-glucose avid lymph nodes seen on positron emission tomography- computed tomography –what looks like cancer may not always be so

**DOI:** 10.1186/1470-7330-14-27

**Published:** 2014-07-31

**Authors:** Anum Imran Malik, Noreen Akhtar, Asif Loya, Muhammed Aasim Yusuf

**Affiliations:** 1 Shaukat Khanum Memorial Cancer Hospital & Research Centre, 7-A, Block R-3, M.A Johar Town, Lahore, Pakistan

**Keywords:** Pakistan, Endoscopic ultrasound, Fine needle aspiration, PET-CT

## Abstract

**Background:**

Patients suffering from malignancies often undergo serial positron emission tomography - computed tomography (PET-CT) scans, using 2-deoxy-2-[^18^F] fluoro-D-glucose (FDG) for diagnosis and follow up. This principle may also be applied to benign conditions as inflammatory cells take up increased amounts of FDG as well. The aim of our study was to retrospectively review the cytological diagnoses made at EUS-FNA of FDG-avid PET-CT lesions in patients with a history of cancer and to determine whether the cause of FDG-avidity was neoplastic or benign.

**Methods:**

We used the endoscopy database to extract clinical information on all patients with malignancies who underwent EUS-FNA to obtain tissue from FDG-avid nodes seen on PET-CT at our institution from 2009 – 2012. All patients who were referred for EUS-FNA after their scans were included. Those who had contraindications to endoscopic procedures were excluded.

**Results:**

The most common location of positive lymph nodes was the subcarinal region (46%). A definitive diagnosis was obtained in 87.8% cases, of which 51.2% had a diagnosis of malignancy confirmed on cytology, while 36.5% were benign. Out of these, 29% had granulomatous inflammation. In 12.2% of cases no definitive diagnosis was obtained.

**Conclusion:**

Our results show that great caution should be exercised when evaluating FDG-avid PET-CT nodes in patients with known malignant disease, as a significant proportion of these lesions may be benign, particularly in geographic locations with a high background prevalence of granulomatous inflammation.

## Background

Patients suffering from malignant neoplasms will often undergo serial positron emission tomography - computed tomography (PET-CT) scans, using 2-deoxy-2-[^18^F] fluoro-D-glucose (FDG), for initial diagnosis, follow up during treatment, and to detect relapse [1]. The increased uptake of FDG in neoplastic lesions, due to their high glycolytic activity, makes PET-CT an extremely useful tool in the diagnosis and follow-up of malignancy [1]. However, this same principle may also be applied to the diagnosis and monitoring of benign conditions [1-5]. It is postulated that inflammatory cells and mediators take up increased amounts of FDG, leading to high standard uptake values (SUV) in the setting of infection and inflammation [1,3,4]. This can often lead to false positive results in malignancy, where inflammation or infection may be mistaken for active malignant disease [4]. In countries with a high prevalence of granulomatous inflammation, such as tuberculosis, distinguishing between the two becomes even more important. Such areas include large parts of Asia and Africa, which have a very high prevalence of tuberculosis [6]. 22 countries are responsible for 80% of the global tuberculosis burden, of which Pakistan is the fourteenth [7]. Endoscopic ultrasound guided fine needle aspiration (EUS-FNA) provides a useful and low risk means of obtaining cytological samples from lesions seen at PET-CT and distinguishing between malignancy and other causes of FDG-avidity, such as infection or inflammation [5,8].

The aim of our study was to retrospectively review the cytological diagnoses made at EUS-FNA of FDG-avid PET-CT lesions in patients with a known history of malignant neoplasms and to determine whether the cause of FDG-avidity was a neoplastic or a benign/inflammatory process.

## Methods

We used the endoscopy database to extract information on all patients with malignant neoplasms who underwent EUS-FNA of FDG-avid nodes seen on PET-CT scan at our institution from 2009 – 2012. We included all patients who were referred for EUS-FNA after their PET-CT scans. The patients who were excluded were those who had contraindications to endoscopic procedures, including but not limited to, coagulopathy, gastrointestinal tract obstruction preventing access to the area of interest and inability to open the mouth, preventing insertion of the echo-endoscope. Clinical information, imaging findings, EUS findings and cytopathology results including the results of rapid on-site evaluation (ROSE) were collected. Lymph nodes seen on PET-CT were considered to be FDG-avid if the standard uptake value (SUV) was > 2.5.

After approval from the institutional review board, a total of forty-one patients were selected for this study. All PET-CT scans were done on a Philips Gemini TF 16 slice multi-detector computed tomography scanner. The dose of FDG F18 to be administered was determined according to the weight of the patient, and ranged from 300-450 MBq for a weight range of 60 kg to 100 kg. Scans were performed approximately 50 minutes after administration of FDG. Whole-body CT scan was performed initially, starting from the skull vertex to the mid-thighs with contiguous slices of 5 mm thickness. Depending upon the clinical indication, intravenous contrast was also administered. After CT, PET data was acquired with the patient lying absolutely still in the same position. The data was acquired starting from the thighs and proceeding towards the head, that is, in a direction opposite to that for CT image acquisition. Typically, 6-8 bed positions were taken with 2-5 minutes acquisition at each position, depending on the patient’s body mass index (BMI). All data obtained is corrected for dead time, scatter, random counts and attenuation, automatically. The threshold value of metabolic index was set at 2.5 because this is the approximate physiologic background uptake of FDG in the body (range: 0.5 to 2.5), and is usually calculated using the liver as a reference point [9]. The standard uptake value, or SUV, is a semi-quantitative assessment of the radiotracer from a static PET image. It is calculated using the formula below:

$$\mathrm{tracer}\;\mathrm{activity}\;\mathrm{in}\;\mathrm{tissue}/\left(\mathrm{injected}\;\mathrm{dose}/\;\mathrm{patient}\;\mathrm{weight}\right).$$

We measured the maximum SUV in any region of interest. If hypoglycemic agents were used, in diabetic patients, then a glucose-corrected SUV was used, calculated using standard formulae.

Written informed consent for EUS-FNA was obtained from all adult patients or from legal guardians of those below the age of eighteen. All procedures were carried out under conscious sedation, using midazolam and pentazocine, except in four paediatric patients, aged 4 – 14 years, in whom the procedure was performed under general anesthesia. EUS was carried out using an Olympus linear array echo-endoscope, and EUS-FNA was performed using a disposable 22-gauge FNA needle in each case. All procedures were carried out by a single, experienced gastroenterologist. Aspirates were smeared and air-dried, or fixed in 95% alcohol. Using the Diff-Quik stain for ROSE, air dried slides were prepared by a trained cytotechnologist within the endoscopy room and were evaluated immediately by a cytopathologist. Alcohol-fixed slides were later stained using the Papanicolaou stain; samples were also saved for cell-block preparation. ROSE ensured sample adequacy, provided a preliminary diagnosis and helped to decide on the need for immunohistochemical stains. In patients known to have a lymphoproliferative disorder, part of the aspirate was saved in RPMI (Roswell Park Memorial Institute Medium) for subsequent flow cytometry studies.

The institutional review board that approved the study is the Shaukat Khanum Memorial Cancer Hospital & Research Centre Institutional Review Board.

## Results

**Figure 1:**
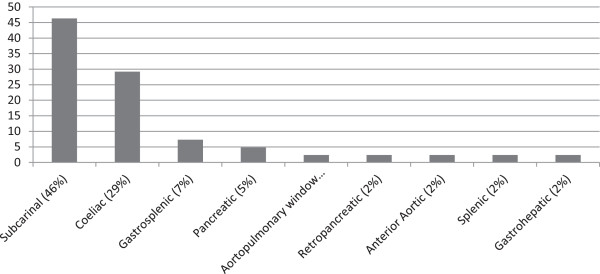
Distribution of FDG avid nodes sampled.

Of the 41 patients studied (age range: 4–78 years; mean age 37.8 years), 14 (34%) were female and 27 (66%) were male. The majority of the patients (n = 24, 58.5%) had an existing diagnosis of classic Hodgkin's lymphoma, followed by diffuse large B-cell lymphoma in 5 (12.2%) (Table 1). Overall, 76% of patients included had lymphoma, while the remainder had a variety of other neoplasms, as one would expect in a tertiary care cancer facility. Of the FDG avid nodes sampled, 19 (46%) were located in the subcarinal region (Figure 1).

**Table 1:** Types of cancers diagnosed

**Type**	**Frequency; (%age) percentage**
Classic Hodgkin’s Lymphoma	24; (58 · 5)
Diffuse Large B-cell Lymphoma	5; (12 · 2)
Ovarian Carcinoma	4; (9 · 7)
Gastrointestinal Stromal Tumour	2; (4 · 9)
Esophageal Carcinoma	1; (2 · 4)
Malignant Melanoma	1; (2 · 4)
Non-Hodgkin’s Lymphoma	1; (2 · 4)
T-cell Lymphoma	1; (2 · 4)
Rectal Adenocarcinoma	1; (2 · 4)
Hepatocellular Carcinoma	1; (2 · 4)
Total:	41; (100)

An average of 2.2 passes were made in order to obtain adequate material for analysis (range: 1–5 passes). A definitive diagnosis was obtained in 36/41 (87.8%) cases, of which 21/41 (51.2%) had a diagnosis of malignancy confirmed on cytology, while 15/41 (36.5%) were benign. Out of these, 12 (29%) had granulomatous inflammation (Table 2). Only one patient out of twelve with granulomatous inflammation was smear-positive for Ziehl-Neelsen stain for acid-fast bacilli. Samples from 9 of these 12 patients were also sent for acid-fast bacilli cultures, all of which were negative.

**Table 2:** Definitive diagnoses obtained at EUS-FNA

**Diagnosis**	**Frequency; (%age)**
**Malignant:**	
Classic Hodgkin’s Lymphoma	13; (31 · 7)
Diffuse Large B-cell Lymphoma	1; (2 · 4)
T-cell Lymphoma	1; (2 · 4)
Ovarian Carcinoma	3; (7 · 3)
Rectal Adenocarcinoma	1; (2 · 4)
Gastrointestinal Stromal Tumour	2; (4 · 8)
Total malignant	21; (51 · 2)
**Benign:**	
Granulomatous Inflammation	12; (29 · 2)
Reactive lymph node	3; (7 · 3)
Total Benign	15; (36.5)
Total (Definitive Diagnosis)	36; (87.8)
**Inconclusive**	5; (12.1)
Total (Overall)	41; (100)

In 5 patients, the results of EUS-FNA were inconclusive, a figure which included two patients in whom the FNA material showed necrosis only.

Cytopathology results were also reviewed. Three patients with lymph nodes showing metastatic ovarian carcinoma (diagnosed as metastatic adenocarcinoma) showed groups and sheets of atypical cells having high nucleus to cytoplasm ratio, overlapping and pleomorphic vesicular to hyperchromatic nuclei with inconspicuous to rarely conspicuous nucleoli, ill defined cytoplasmic borders and cytoplasmic vacuolations. Metastatic rectal adenocarcinoma had groups of atypical columnar cells forming acini/glands with pleomorphic nuclei and necrosis in the background. Granulomatous inflammation was diagnosed by observing scattered loose aggregates of epithelioid histiocytes, few giant cells and rarely necrosis with mixed inflammatory cells in the background. Reactive lymph nodes showed a heterogeneous population of lymphoid cells in different stages of maturation and anthracotic, pigment-laden macrophages in a few cases. No atypical lymphoid cells were seen in these smears. In nodes considered positive for classic Hodgkin’s lymphoma, a heterogeneous population of lymphoid cells, macrophages and rare eosinophils were seen with atypical mononuclear and bi-nucleated lymphoid cells showing large prominent nucleoli. Lymph nodes with FNA diagnoses of diffuse large B-cell lymphoma showed population of large cells with conspicuous nucleoli, high nuclear-cytoplasmic ratio and increased mitotic activity. FNA of GIST showed spindled cells showing mild to moderate nuclear atypia and inconspicuous nucleoli.

Immunohistochemical stains were used on 17 (41%) samples. 11 (64%) samples showed CD30 positive atypical mono- and bi-nucleated cells confirming the diagnosis of classic Hodgkin’s lymphoma. One sample was positive for both CD3 and CD45 (leukocyte common antigen) and negative for CD20, confirming T-cell lymphoma. CDX2 positivity (rectal adenocarcinoma) and CD117 positivity (GIST) was seen in one sample each. We found two samples to be negative for CD15, these were confirmed as classic Hodgkin’s lymphoma on the basis of CD30 positivity.

Nine patients had FDG-avid lymph nodes biopsied to check for residual disease. Of these, 3/9, or 33.3%, were due to benign processes. Granulomatous inflammation was seen in all three of these patients. Of the other six cases, four had residual cancer, while EUS-FNA sampling was inconclusive in two patients, one of which had necrosis on aspirate.

28 patients were found to have FDG-avid lesions during imaging for regular follow-up of neoplasia. 17/28 were found to have relapse of cancer, 9/28 (32%) had benign disease, with granulomatous inflammation in eight and reactive lymphadenopathy in one. In 2/28 patients, cytology results were inconclusive.

3/4 patients who underwent EUS-FNA of FDG avid lesions as part of their initial staging process had benign cytological diagnoses – reactive lymph nodes in two patients, and granulomatous inflammation in one patient. Inconclusive results, as demonstrated by necrotic changes, were seen in the fourth patient.

Four patients had previously received radiotherapy to the area from which EUS-FNA was to be performed. Three of these four patients had received radiotherapy to the chest and now presented with FDG-avid subcarinal nodes in two patients and an FDG-avid aorto-pulmonary window node in one patient. The fourth of these patients had received upper abdominal radiotherapy and now presented with FDG-avid coeliac nodes. All four of these patients had relapsed malignant disease on biopsy of the nodes of interest. Lymphoma was diagnosed in 3 patients whereas 1 patient had adenocarcinoma.

3 patients had a prior history of tuberculosis and 2 of these had granulomatous disease on biopsy of FDG avid nodes while 1 had reactive inflammatory findings.

## Discussion

Patients suffering from malignant disease are usually required to undergo imaging investigations not only for diagnosis but also regularly during treatment and as part of regular follow up. This latter process often involves many years of careful surveillance following treatment for recurrent disease [5]. Additionally, symptoms suggesting recurrence may warrant further imaging and other studies. The assessment of lymph nodes using CT currently relies on the size and appearance of the node, rather than on its functional characteristics. On CT, a normal lymph node is typically <1 cm in size, with smooth and well-defined borders, a central fatty hilum and a homogeneous density. Typically, nodes which are larger than 1 cm in diameter, with a rounded appearance and heterogeneous density are considered to be malignant, especially when multiple such nodes are seen. CT therefore relies on somewhat insensitive size and morphologic criteria and is less accurate than PET-CT in characterizing lymph nodes. This is because not all enlarged nodes are malignant, and small or non-enlarged nodes may still harbour malignancy [10].

However, inflammation can mimic malignancy on PET-CT and it is not possible, at present, to distinguish between these using imaging criteria alone [11]. This sometimes leads to patients being mislabeled as having metastatic or recurrent disease [5]. For this purpose, it is imperative that FDG-avid lesions seen on PET-CT scans are interpreted correctly.

In our review we found that 36.5% of patients with FDG-avid lymph nodes on PET-CT, all of whom had either active neoplasia or a past history of malignancy, actually had benign lesions when these nodes were subjected to EUS-FNA. The most common benign disease seen in this situation was granulomatous inflammation. Quite obviously, this has major implications on patient staging, treatment and prognosis. All patients included in the study had a pre-existing diagnosis of malignancy. The indication for the PET-CT was, in the main, for follow up after treatment, to assess disease response or to check for residual disease.

Our results are comparable to those of Fritscher-Ravens et al. who reported 21 (41.17%) benign lesions out of a total of 51 adequate samples from patients with cancer who were suspected of recurrence [5]. However, Fritscher-Ravens et al. only focused on mediastinal lesions whereas we sampled lesions from multiple locations [5]. To our knowledge, ours is the first study that has included FDG-avid PET-CT lesions from multiple sites.

Another study on lung cancer, by Eloubeidi et al., found the rate of benign lesions to be as high as 64% [12]. Their higher rate of positivity, however, is likely to be because they included patients with suspected lung cancer in their study, whereas in our study all patients had a confirmed background diagnosis of cancer. Fritscher-Ravens et al. found the majority of benign results to be due to inflammatory changes, with granulomatous inflammation following close behind [5]. In comparison, we found a much higher percentage of granulomatous inflammation in our patients, (12/15 benign lesions). This is likely to reflect the high background prevalence of tuberculosis in this part of the world. However, despite the high rate of granulomatous inflammation in our cohort, only one sample was positive on Ziehl-Neelsen stain, which is probably due to the known low sensitivity of this stain. We do not routinely use other stains, such as auramine-rhodamine or immunofluorescence for AFB to detect AFB at our institution*.* 9 out of 12 patients with granulomatous inflammation had cultures for AFB done, all of which were negative. In contrast with the work of Fritscher-Ravens et al. we found a much lower proportion of patients with reactive lymph nodes (3/15) [5].

We classified necrotic changes on cytopathology as inconclusive results because excisional biopsies were not done and it cannot be said with certainty if the necrosis was limited to only the area that was sampled or affected the entire node.

False-positive FDG-avid PET-CT lesions have mainly been reported in cases of granulomatous processes such as tuberculosis and sarcoidosis [4,13]. This makes our results relevant not only for regions where tuberculosis is widespread, such as South Asia and Africa, but also for other parts of the world with large immigrant populations derived from these areas. Therefore, extreme caution should be exercised when interpreting positive PET-CT results in these patients. A similar conclusion was reached by Goo et al. [2]. We feel that oncologists and others treating cancer patients who regularly obtain PET-CT studies on their patients as part of this process need to be aware of these findings. While EUS-FNA was only performed on FDG-avid nodes, one cannot completely rule out the possibility of a node which is negative on PET-CT (i.e. non - FDG-avid) still harbouring a focus of malignancy.

We found that 3/9 patients who were sampled during treatment had benign conditions on cytology, all of which were granulomatous inflammation. For patients who underwent EUS-FNA during their follow-up period, 9/28 (32.1%) had benign lesions, of which eight were granulomatous inflammation. These are significant false positive rates for lesions that mimic cancer, in the setting of cancer, but are in fact benign lesions. Without EUS-FNA, these patients might well have been assumed to have residual cancer, and been subjected to further, potentially hazardous, treatment.

Schaefer et al. and Crocchiolo et al. discussed in their reports that 10-40% of FDG avid nodes on PET-CT, thought to be malignant, actually turn out to be benign [14,15]. In keeping with this, we found that 32.4% (12/37) of patients with known malignancy, now post-treatment, had benign results on biopsy of FDG-avid nodes seen on PET-CT. Our review may have been limited by the small sample size. Also, since two different cytopathologists evaluated our specimens, there may have been subjective variation in the interpretation of the findings. We were not limited by inadequate sampling, however, since we achieved a high rate of definitive diagnosis (92.6%), with an average of 2.2 passes.

Our review demonstrates that extreme caution should be exercised when evaluating FDG-avid lymph nodes on PET-CT in patients with known malignant disease, as a significant proportion of FDG-avid lesions may be benign. In our series, 36.5% had non-malignant conditions, with granulomaotus inflammation being the most common non-malignant lesion seen. Our results are of particular significance to physicians practicing in settings where granulomatous inflammatory diseases are widespread and also of importance for oncologists, and other physicians, practicing elsewhere in the world, who regularly deal with patients from these areas. These results have the potential to significantly impact patient diagnosis, staging and subsequent management. Many patients will be able to discontinue cancer treatment sooner, or to avoid unnecessary re-treatment, as a result of an appropriate diagnostic intervention such as EUS-FNA, when FDG-avid nodes are seen on PET-CT scan. This has significant impact in improving quality of life in these patients and all physicians treating such patients need to be aware of these important findings.

## Conclusion

Our results show that great caution should be exercised when evaluating FDG-avid lymph nodes on PET-CT in patients with known malignant disease, as a significant proportion of these lesions may be benign. Failure to establish a tissue diagnosis in such a situation can lead to patients being treated inappropriately, with potential for harm to the patient. We recommend that all such lesions need to be sampled and an accurate diagnosis made prior to instituting, or making changes to, therapy. EUS-FNA, with rapid on-site evaluation offers an optimal means of sampling such lesions.

## Abbreviations

PET-CT: Positron emission tomography-computed tomography; DG: 2-deoxy-2-[^18^F] fluoro-D-glucose; SUV: Standard uptake value; EUS-FNA: Endoscopic ultrasound-fine needle aspiration; ROSE: Rapid on-site evaluation; RPMI: Roswell park memorial institute medium; GIST: Gastrointestinal stromal tumour; AFB: Acid fast bacilli.

## Competing interests

The authors declare no competing interests.

## Authors’ contributions

MAY conceived the study, participated in data collection and in writing of the first draft of the manuscript. He was also responsible for review of the manuscript. AIM participated in data collection and in writing of the first draft of the manuscript. She also participated in review of the manuscript. NA and AL participated in writing and review of the manuscript. All authors read and approved the final manuscript.
